# What is Going on in Psychiatry When Nothing Seems to Happen?

**DOI:** 10.3389/fpsyt.2013.00178

**Published:** 2013-12-27

**Authors:** Adonis Sfera

**Affiliations:** ^1^Department of Psychiatry, Patton State Hospital, Patton, CA, USA

**Keywords:** neurobiology, CMV, de novo mutations, optogenetics, two photon laser scanning microscopy

When I started my career in psychiatry, more than 20 years ago, nobody thought about cognitive impairment in schizophrenia, we thought of this disorder in terms of hallucinations and delusions. The prevailing idea was that of a “chemical imbalance” somewhere in the brain that one could correct with dopamine blockers. If we could only keep that dopamine down, we thought, patients with schizophrenia could live a normal life. Comparisons with hypertension and diabetes were ubiquitous, thus my generation built “clean” and “dirty” drugs that carpet bombed receptors far and near monoamine tracts in the brain. These drugs were blunt and worked on the receptors in sledgehammer-like fashion. Yet, there was a lot of optimism in the air, we were talking recovery and cure. The working hypothesis was something like this: our patients accidentally fell from the bicycle of life and bruised their knees. Our job was to bandage those knees and help them back on the bicycle after which they would live happily ever after.

Fast forward to the present day: medications do indeed help acute symptomatology of schizophrenia, but do not make a dent in incidence, cognitive symptoms, or disability. This year alone 100,000 young people will have a first episode of schizophrenia and 5% of them will die by suicide. Sustained recovery is <14% within the first 5 years following a psychotic episode. In the past 3–4 years the new psychotropic drugs’ pipeline became dry. There are multiple reasons for this state of affairs, but the main one is the lack of success with new mechanisms of action of antipsychotic and antidepressant drugs as well as lack of viable, measurable biological markers. In terms of new drugs for our patients, there will be few in the coming years. I believe that we are in a stagnation period that will probably last a decade or more.

## Meanwhile Psychiatry is Gradually Becoming a Branch of Clinical Neuroscience

Many psychiatrists do not agree with this trajectory for our field. Just like the psychiatrists of the previous generation felt comfortable in psychoanalysis, some psychiatrists today feel that they are experts in psychopharmacology by memorizing details about the interaction of drugs with neurotransmitters and receptors. However, many of the same psychiatrists, have little real understanding or concern about what actually might be wrong in the brains of individuals with psychiatric disorders, being content to describe the illness as a “chemical dysfunction.” Moreover it is not clear that any psychotropic drugs in use today have anything to do with the etiology or pathogenesis of the diseases they are used to treat.

## So What is Happening in Psychiatry When Nothing is Going on?

Here is what is happening in the background: the pause in new drug development is leading to the design of new, finer tools of the trade that are gradually enlarging our understanding of brain physiology and pathology. There is not much talk in the media about the development of these new tools, they are taking shape quietly, behind the scenes in academic settings, but they are already bearing fruit.

## What are the New Tools?

### Visualizing the thoughts (*in vivo* two photon laser scanning microscopy)

The dream of neuroscientists for the past centuries was to be able to see the brain at work *in vivo*. This finally came to fruition by the advent of two photon laser scanning microscopy. This technique allows us to peer in the brain of live mice or cultured brain cells and to record ongoing cellular processes in real time. We are learning vital information on dendritic spines of pyramidal neurons. Dendritic spines: cortical pyramidal neurons, express tens of thousands of small protuberances on their dendritic trees. These dynamic structures receive excitatory information from other neurons, and play a significant role in cognition. It is believed that changes in spine number or morphology precede the cognitive cascade in many neuropsychiatric disorders.

#### Fruits

Patients with schizophrenia, for example, present with decrease in basal dendrites and spines in layers three and five, especially in area nine of the brain. Techniques to measure dendritic spine density are emerging as new biological markers of the disease (1). Novel neuroprotective compounds and techniques (such as electrical stimulation) seem to increase dendrites and spines, especially in the hippocampus.

### Optogenetics

Optogenetics is an *in vivo* technique that came about by combining genetics and optics to control events within brain cells. This is how it works: genes coding for light sensitive or fluorescent proteins are inserted into the mouse DNA. Part of the rodent’s skull is replaced by a transparent material, creating a window through which the brain is visualized. This technique allows scientists not only to see the light sensitive proteins at work in a moving mouse, but also to switch on and off (via light) various neural circuits.

#### Fruits

Using optogenetics, scientists were able to manipulate EEG gamma oscillations (~40 Hz) thought to be crucial for cognition in schizophrenia. Optogenetics has also been combined with fMRI. Optogenetic functional magnetic resonance imaging (ofMRI) is a novel approach that combines optogenetic control of neural circuits with high-field functional MRI, creating a powerful tool that can monitor and influence neural activity arising from specific circuits in any area of interest (2).

### “I cannot see a string, but I can see a cable” (white matter tractography)

We can see individual neurons (in cultures or brain) by microscopy only, they cannot be seen in real time *in vivo* in the brain.

Why not? The best MRI machines now-a-days have a resolution of about 1 mm. The problem is that 1 mm of brain tissue contains about 1,000 neurons.

What about axons? The good news is that axons travel together forming thick cables. Large cables, like corpus callosum, can be seen by naked eye. Thinner ones (more than 1 mm) can be picked by MRI and thus can be seen *in vivo* in real time. That is how white matter tractography originated. The human connectome project is a very ambitious plan of mapping the entire white matter (“cables” that connect brain regions) in healthy and diseased brains. Wernicke (3) was the first to posit that abnormal connections among brain regions may play a critical role in the etiology of schizophrenia.

#### Fruits

It is currently believed that schizophrenia is a neurodevelopmental brain dysconnection syndrome, involving abnormal interactions between widespread brain networks. Accumulating evidence from a growing number of neuroimaging studies have begun to identify heritable biomarkers of this complex disorder. Current studies demonstrate both reduced and enhanced connectivity among several brain areas, including dorso-lateral prefrontal cortex (DLPF) with the temporal lobe, parietal lobe, hippocampal formation, thalamus and cerebellum, cingulate cortex, and also limbic regions (particularly amygdala-frontal connections) (4). There are novel rehabilitation techniques that seem to help the brain “rewire” to a certain extent during adult life.

### Schizophrenia in a petri dish (cultured neurons)

Neuropsychiatric disorders can be studied more effectively thanks to a new method for obtaining mature neurons from reprogramed skin cells. Researchers can take skin cells from living patients and convert them into neurons or astrocytes that mimic the activity of the patient’s own brain cells. These studies have demonstrated the collaborative role between neurons and astrocytes in human cognition. Astrocytes participate in glutamatergic tripartite synapses that support cognitive functions, such as learning, perception, conscious integration, memory formation, and retrieval.

#### Fruits

In the past few years the classical view of astrocytes as a simple supportive cell for neurons, has been replaced by a new vision in which glial cells are active elements of the brain. For example, neurons grown *in vitro* along with astrocytes differentiate more rapidly and form many more synapses than neurons grown alone. This raises the possibility that astrocytes may be as important, or even more important than neurons, not only during brain development, but also in its day to day function and cognition. Targeting astrocyte receptors and pathology is rapidly becoming a novel area of interest in cognitive disorders (5).

### Gazing into the crystal brain (CLARITY)

Another tool discovered recently is called CLARITY. CLARITY is an acronym, for Clear Lipid-exchanged Anatomically Rigid Imaging/immunostaining-compatible Tissue hydrogel. By replacing the brain’s fat with a clear gel, CLARITY turns the opaque and impenetrable brain into a transparent and permeable structure. This postmortem technique allows us to look into the human brain like into a crystal ball and identify cells and tracts with utmost precision.

#### Fruits

This postmortem technique is complementary to the *in vivo* human connectome project. It can microscopically zoom in and provide details that fMRI may miss because of limited resolution (6).

### You are never alone, you always have your germs (the human microbiome)

The microbial world of our bodies was recently mapped by a consortium of scientists. The results show that our bodies are complex ecosystems in which human cells represent only 10% of the population. These results have altered how we think about what it means to be human.

#### Fruits

How our microbial world influence the development of brain and behavior will be one of the great frontiers of clinical neuroscience in the next decade (7).

### If it is not nature it must benurture (mapping the epigenome)

All the cells of our body have exactly the same genes, yet a skin cell, for example, is very different from a muscle fiber or a neuron. Why? Because only the pertinent genes in each cell are allowed to be expressed, while the others are silenced by the epigenome. Epigenetics refers to the study of stable, often heritable, changes that influence gene expression that are not mediated by DNA sequence. Multiple disease processes, including cancer, are now well known to be associated with characteristic alterations in the epigenome, such as the patterns of chromatin, DNA methylation, and gene expression. The recently launched NIH Roadmap Epigenomics Mapping Consortium, provides a public resource of human epigenomic data for use in basic biology and disease-oriented research. This project is complementary to ENCODE (ENCyclopedia of DNA Elements) launched in September 2003, to identify the human genome sequence.

#### Fruits

Until recently, research has primarily focused around the role of genetic factors in schizophrenia, but other variables, including the environment, were found to be as important in the onset of the disease. For example, genetic variation alone cannot explain why identical twins often differ, with one twin developing schizophrenia, and the other being free of the condition. A promoter on chromosome 17q25.1 called ST6GALNAC1 was found to differ in identical twins discordant for schizophrenia. In the future this new knowledge may be used to develop a blood test to diagnose patients with schizophrenia (8).

### What does the brain do when it does nothing? (low frequency oscillations and DMN)

Recent functional magnetic resonance imaging studies have identified structured patterns of slow neuronal oscillations in the resting human brain (0.001–0.1 Hz). It was called default mode network (DMN) and it refers to a group of cortical areas that are most active in the resting state and decreases its activity during the performance of a task. This brain network transcends levels of consciousness, being present during anesthesia, sleep, or coma raising speculations about the origin of what we call self-awareness or consciousness. The DMN was found to differ in schizophrenia patients with poor insight compared to those with good insight (9). Poor insight is believed to reflect decreased connectivity in the DMN.

#### Fruits

A new methodology to enhance brain connectivity (help the adult brain re-“wire”) in schizophrenia was recently described. It is called fMRI-brain-computer interfaces (fMRI-BCI). This novel therapeutic technique allows subjects to achieve self-regulation of various brain regions by getting feed-back via real time fMRI (10).

### *De novo* mutations

Given the significantly reduced fertility of individuals with schizophrenia, why has the prevalence of this disease remained stable over time? Several recent studies have shown that newly arising (*de novo*) mutations contribute to the genetics of schizophrenia. The strongest evidence to date for the association between schizophrenia and *de novo* mutation comes from studies of copy number variation (CNV). What are CNVs? CNVs are microdeletions or microduplications of one or more sections of the DNA on a specific chromosome.

Recent data robustly confirm the association of schizophrenia with 1q21.1, 15q13.3, and 22q11.21 deletions, 16p11.2 duplications, and exonic NRXN1 deletions. However the strongest association with this disease is a deletion in chromosome 3q29 that was previously described in a mild-moderate mental retardation syndrome, autism spectrum disorders, and epilepsy.

#### Fruits

A small CNVat 22q13.31 on schizophrenia affects the gray matter concentration (GMC) in the frontal cortex, demonstrating that CNVs not only have the potential of influencing schizophrenia risk, but also altering GMC in specific brain regions (11).

## References

[1] Somenarain L Jones LB Dendritic and spine alterations in areas 9 and 17 in schizophrenia and Huntington chorea and the role of neuroleptic exposure. Open J Psychiatry (2012) 2:243–8 10.4236/ojpsych.2012.23032

[2] Witt A Palmigiano A Neef A El Hady A Wolf F Battaglia D Controlling the oscillation phase through precisely timed closed-loop optogenetic stimulation: a computational study. Front Neural Circuits (2013) 7:49 10.3389/fncir.2013.00049PMCID 23616748 PMC3627980

[3] Wernicke C Grundriss der Psychiatrie in Klinischen Vorlesungen. Leipzig: Thieme (1894, 1896).

[4] Whitford TJ Kubicki M Shenton ME Diffusion tensor imaging, structural connectivity, and schizophrenia. Schizophr Res Treatment (2011) 2011:709523 10.1155/2011/709523 22937272 PMC3420716

[5] Santello M Calí C Bezzi P Gliotransmission and the tripartite synapse. Adv Exp Med Biol (2012) 970:307–31 10.1007/978-3-7091-0932-8_14 22351062

[6] Chung K Wallace J Kim SY Kalyanasundaram S Andalman AS Davidson TJ Structural and molecular interrogation of intact biological systems. Nature (2013) 497:332–7 10.1038/nature12107 23575631 PMC4092167

[7] Peterson J Garges S Giovanni M McInnes P Wang L Schloss JA The NIH human microbiome project. Genome Res (2009) 19(12):2317–23 10.1101/gr.096651.109 19819907 PMC2792171

[8] Dempster EL Pidsley R Schalkwyk LC Owens S Georgiades A Kane F Disease-associated epigenetic changes in monozygotic twins discordant for schizophrenia and bipolar disorder. Hum Mol Genet (2011) 20(24):4786–96 10.1093/hmg/ddr416PMCID 21908516 PMC3221539

[9] Yu Q Allen EA Sui J Arbabshirani MR Pearlson G Calhoun VD Brain connectivity network in schizophrenia underlying resting state functional magnetic resonance imaging. Curr Top Med Chem (2012) 12(21):2415–25 10.2174/156802612805289890 23279180 PMC4429862

[10] Ruiz S Birbaumer N Sitaram R Abnormal neural connectivity in schizophrenia and fMRI-brain-computer interface as a potential therapeutic approach. Front Psychiatry (2013) 22:17 10.3389/fpsyt.2013.00017 23525496 PMC3605516

[11] Liu J Ulloa A Perrone-Bizzozero N Yeo R Chen J Calhoun VD A pilot study on collective effects of 22q13.31 deletions on gray matter concentration in schizophrenia. PLoS One (2012) 7(12):e52865 10.1371/journal.pone.0052865 23285208 PMC3532105

